# Deep Learning-Based Semantic Segmentation for Objective Colonoscopy Quality Assessment

**DOI:** 10.3390/jimaging11030084

**Published:** 2025-03-18

**Authors:** Radu Alexandru Vulpoi, Adrian Ciobanu, Vasile Liviu Drug, Catalina Mihai, Oana Bogdana Barboi, Diana Elena Floria, Alexandru Ionut Coseru, Andrei Olteanu, Vadim Rosca, Mihaela Luca

**Affiliations:** 1 Institute of Gastroenterology and Hepatology, “Grigore T. Popa” University of Medicine and Pharmacy, 700111 Iasi, Romania; radu-alexandru.vulpoi@umfiasi.ro (R.A.V.); vasile.drug@umfiasi.ro (V.L.D.); catalina.mihai@umfiasi.ro (C.M.); oana.barboi@umfiasi.ro (O.B.B.); diana-elena.floria@umfiasi.ro (D.E.F.); alexandru-ionut.coseru@umfiasi.ro (A.I.C.); vasile-andrei.olteanu@umfiasi.ro (A.O.); vadim.rosca@umfiasi.ro (V.R.); 2 Institute of Computer Science, Romanian Academy, Iasi Branch, 700481 Iasi, Romania; mihaela.luca@iit.academiaromana-is.ro

**Keywords:** automatic annotation, Boston Bowel Preparation Scale, colonoscopy quality evaluation, color features, deep learning, semantic segmentation

## Abstract

**Background:** This study aims to objectively evaluate the overall quality of colonoscopies using a specially trained deep learning-based semantic segmentation neural network. This represents a modern and valuable approach for the analysis of colonoscopy frames. **Methods:** We collected thousands of colonoscopy frames extracted from a set of video colonoscopy files. A color-based image processing method was used to extract color features from specific regions of each colonoscopy frame, namely, the intestinal mucosa, residues, artifacts, and lumen. With these features, we automatically annotated all the colonoscopy frames and then selected the best of them to train a semantic segmentation network. This trained network was used to classify the four region types in a different set of test colonoscopy frames and extract pixel statistics that are relevant to quality evaluation. The test colonoscopies were also evaluated by colonoscopy experts using the Boston scale. **Results:** The deep learning semantic segmentation method obtained good results, in terms of classifying the four key regions in colonoscopy frames, and produced pixel statistics that are efficient in terms of objective quality assessment. The Spearman correlation results were as follows: BBPS vs. pixel scores: 0.69; BBPS vs. mucosa pixel percentage: 0.63; BBPS vs. residue pixel percentage: −0.47; BBPS vs. Artifact Pixel Percentage: −0.65. The agreement analysis using Cohen’s Kappa yielded a value of 0.28. The colonoscopy evaluation based on the extracted pixel statistics showed a fair level of compatibility with the experts’ evaluations. **Conclusions:** Our proposed deep learning semantic segmentation approach is shown to be a promising tool for evaluating the overall quality of colonoscopies and goes beyond the Boston Bowel Preparation Scale in terms of assessing colonoscopy quality. In particular, while the Boston scale focuses solely on the amount of residual content, our method can identify and quantify the percentage of colonic mucosa, residues, and artifacts, providing a more comprehensive and objective evaluation.

## 1. Introduction

The acquisition of knowledge through visual information is typically categorized into object detection and localization (marked by bounding boxes or contours), image categorization (or classification), or semantic segmentation (which operates at the pixel level). Semantic segmentation involves dividing an image into regions with semantic significance by assigning a class label to each pixel. Specifically, the method proposed in this study provides detailed information about key regions of interest (e.g., intestinal mucosa). In contrast, classic segmentation generally focuses on forms and shapes that define well-known anatomical structures (e.g., polyps) which are typically used for diagnostic purposes [1,2].

### 1.1. Evolution of Semantic Segmentation in Medical Imaging

The evolution of semantic segmentation has progressed in parallel with advancements in machine learning. Early methods for semantic segmentation depended on manual effort and algorithms guided by pre-defined criteria. These methods included techniques such as thresholding, clustering (e.g., k-means), and edge detection, which were often insufficient when considering complex scenes.

In recent decades, new methods have started incorporating feature-based techniques [3]. Algorithms extracting features have used colors [4], textures [5,6], and shape features to classify pixels; however, these methods have not attained the desired accuracy and generalization metrics. In this context, convolutional neural networks (CNNs) [7] marked a significant turning point. In 2012, AlexNet [8] demonstrated the effectiveness of deep learning (DL) in image classification, leading to interest in its application for segmentation tasks. In 2015, fully convolutional networks (FCNs) were introduced, facilitating DL for semantic segmentation and outputting spatial information in terms of pixel-wise class predictions. Techniques such as dilated convolutions [9] have been developed to increase the receptive field without loss of resolution; meanwhile, multi-scale segmentation methods emerged to capture contextual information at different scales, thus improving the segmentation quality. More recent advancements have incorporated attention mechanisms to help models focus on relevant parts of the image, further enhancing segmentation performance. Models such as DeepLab [10,11] use attention to refine the segmentation results through consideration of the global context.

### 1.2. The Need for AI-Driven Colonoscopy Quality Assessment

The motivation for employing automated semantic segmentation techniques in colonoscopy evaluations resides in the fact that human assessments of the same colonoscopy frame can vary significantly (e.g., due to factors such as experience or fatigue). The presence of such variability [12] may lead to missed lesions or inconsistencies in diagnostic quality, directly impacting patient outcomes. Artificial intelligence (AI)-driven segmentation tools increase the potential for standardizing and improving the accuracy of diagnostics, thus reducing subjectivity in the diagnostic process [13].

Proper preparation of the digestive tract is essential to ensure the success of a colonoscopy and obtain reliable results. Compliance with the recommended colonoscopy preparation guidelines [14] ensures a clear view of the intestinal mucosa, facilitating the identification and assessment of any lesions or abnormalities. At present, the responsibility for evaluating the degree of bowel preparation rests with the physician during the colonoscopy. The decision to proceed with the investigation or to discontinue and reassess the patient after another bowel preparation regimen is based both on the physicians’ experience and their degree of subjectivity. To reduce subjectivity, several scoring systems have been developed to evaluate the quality of bowel preparation for colonoscopy [15]. These scales have been approved for use in colonoscopy, with some—such as the Boston Bowel Preparation Scale (BBPS)—being widely used in clinical practice [16]. However, the results remain operator-dependent and vary significantly between users. Factors such as personal definitions of cleanliness, the effort and fatigue associated with the washing process, and reliance on the operator’s memory can influence the final score [17].

The implementation of external objective operator-independent systems, such as a deep learning-based AI system, may ensure a constant improvement in the quality of performed colonoscopies, contributing to the early detection of lesions [18], standardization of quality metrics [19], and an overall improvement in diagnostic accuracy during colonoscopies, thus ensuring consistent and reliable outcomes for patients [20].

### 1.3. Study Objectives

In this study, we develop a deep learning-based AI system using semantic segmentation techniques for automated colonoscopy quality assessment. Our approach aims to quantify key regions of interest (i.e., the intestinal mucosa, residues, lumen, and artifacts) and provide a more comprehensive and objective evaluation, compared to the BBPS.

**Figure 1:**
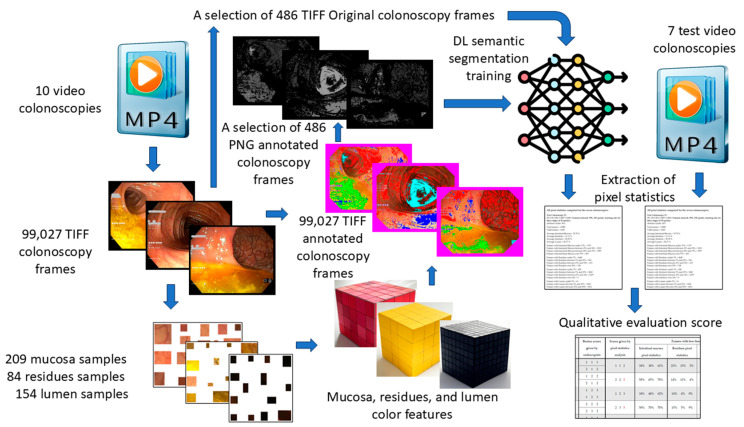
Schematic data flow from video colonoscopies to qualitative evaluation score.

A schematic representation of our workflow, from colonoscopy video acquisition to quality assessment, is provided in Figure 1.

The remainder of this paper is organized as follows. Section 2 describes the methodology, detailing the acquisition of the dataset, annotation process, and the design of the deep learning-based semantic segmentation network. Section 3 presents the results obtained when applying the proposed AI model, comparing its performance with traditional color-based segmentation methods and analyzing its effectiveness in assessing the colonoscopy quality. Section 4 discusses the findings in relation to the existing literature, highlighting the implications for clinical practice, potential improvements, and the study’s limitations. Finally, Section 5 concludes the study by summarizing its key contributions and outlining future research directions.

## 2. Materials and Methods

This section details the design and implementation of our AI system, the acquisition and preparation of colonoscopy video data, the annotation process for training the model, and the evaluation framework used to measure the system’s performance.

### 2.1. The Set of Colonoscopies Used for Training

For this study, video colonoscopies were selected from the database of the St. Spiridon University Hospital in Iași. The selected videos were obtained in patients who underwent colonoscopy for colorectal cancer screening (at least 50 years old; asymptomatic or with positive fecal immunohistochemical test, FIT). The included videos were evaluated by two colonoscopy experts, following consensual colonoscopy protocols. Multiple colonoscopy systems were used for image acquisition, including the OLYMPUS OPTERA CV-170, OLYMPUS EXERA III CV-190, and OLYMPUS X1 CV-1500. The video files were edited to include only the withdrawal phase, excluding segments involving therapeutic endoscopic interventions such as polypectomies, biopsies, and hemostasis procedures. This approach aligned with our database’s primary objective: evaluating the overall quality of a colonoscopy, including bowel preparation, identification of colonic anatomical landmarks, and proper visualization of the intestinal mucosa.

During the analysis of the recordings, certain artifacts that could interfere with adequate mucosal examination were noted. These artifacts included light reflections on the mucosa, blurry images, colonoscopy system error messages displayed on-screen and, occasionally, recorded metadata overlapping the video footage. These factors were identified as potential challenges for achieving consistent and accurate evaluations. We used 10 screening colonoscopies, saved as Standard Definition (SD) **.avi** files during the colonoscopy examination. To be able to process them in Matlab 2023b, we transformed them into **.mp4** files. Then, each colonoscopy file was decomposed into frames with dimensions of 720 × 576 pixels (RGB color-coded with 24 bits/pixel) as **TIFF** files, resulting in a total of 109,163 images. All the routines developed for the method presented in this paper were implemented as Matlab scripts.

### 2.2. Regions of Interest

Our goal was to characterize colonoscopy frames by dividing them into four key regions of interest (ROIs): The intestinal mucosa, residues, lumen, and artifacts. A frame dominated by well-visualized intestinal mucosa suggests that the endoscopist could effectively assess it, while frames containing significant residues indicate inadequate bowel preparation. Frames showing the lumen suggest proper camera positioning to visualize all intestinal walls. Conversely, frames dominated by artifacts are considered uninformative or unusable. For this purpose, we classified each pixel in a colonoscopy frame into one of these four regions using deep learning-based semantic segmentation, enabling a description of each frame as a percentage combination of pixels pertaining to these regions.

### 2.3. Region Color Features

**Figure 2:**
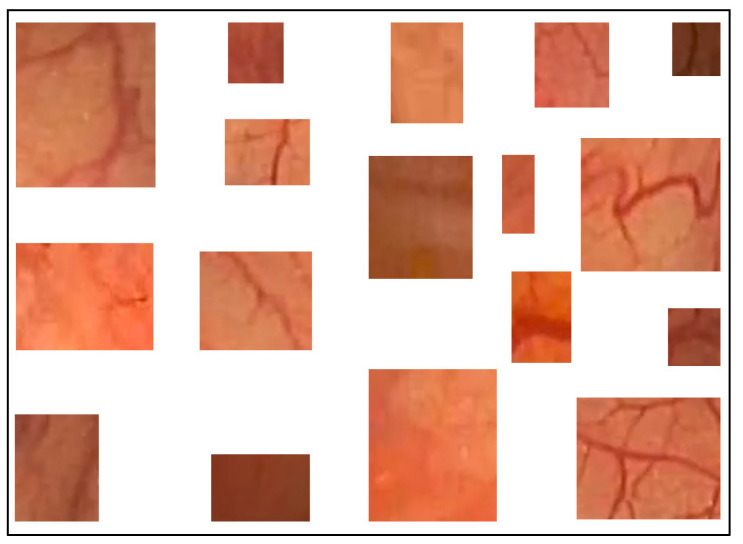
Examples of intestinal mucosa image samples used to generate color features.

**Figure 3:**
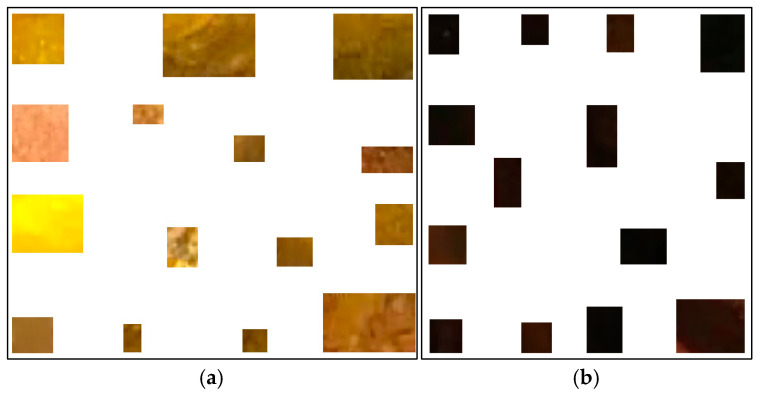
Examples of residues (**a**) and lumen (**b**) image samples used to generate their color features.

Previously, we proposed a method to classify pixels pertaining to the intestinal mucosa, residues, and artifacts in a colonoscopy frame based on their color contents [4]. The pixels pertaining to intestinal mucosa are mostly in shades of red to brown. Residues are mostly colored from yellow to brown, while artifacts are mostly white or certain saturated colors (e.g., orange, green, or blue). In order to capture all pixel color nuances inherent to the intestinal mucosa, we developed a routine that helps a user to parse colonoscopy frames. When the mucosa is clearly seen, it allows the user to mark, extract, and save a small rectangular area of the image which best describes the mucosa in terms of its color nuances. We used 209 intestinal mucosa image samples (examples are shown in Figure 2) to obtain the results presented in this paper. The same routine was used to develop a collection of 84 image samples reflecting residues (see Figure 3a). While we had image samples collected for artifacts and lumen in the beginning, this approach was not sufficiently productive in either case (we still used 154 image samples for the lumen; see Figure 3b). Thus, in these cases, we found other solutions that will be explained later in this paper.

In our approach, color features are represented as a matrix of dimensions 256 × 256 × 256, where each of its elements signals the presence or absence of a color nuance in a colonoscopy frame. Such a matrix can deal with a maximum of 16,777,216 nuances. However, our colonoscopy frame regions include just a few nuances, with the rest of the positions being zero. While something like the ***RGB*** (Red, Green, Blue) color cube could be used in this context, we chose to work with the ***Lab*** cube (corresponding to the CIELAB color system) [21]. To obtain three different color features for the intestinal mucosa, residues, and lumen, we developed another routine. In the case of the intestinal mucosa, all the image samples were saved as RGB TIFF files in the same folder. This routine reads all these files sequentially, and transforms them from ***RGB*** to ***Lab***. For each pixel in these images, the coefficients ***L***, ***a***, ***b*** (e.g., 118, 129, 143) corresponding to its nuance were taken and the corresponding element of the color feature ***Lab*** cube was increased by 1. In this way, all the nuances pertaining to the intestinal mucosa were captured, some of them more than once. Theoretically, the nuances that appear more frequently are more characteristic of the intestinal mucosa. However, the number of different nuances taken into consideration depends on how many image samples are collected and how different the colonoscopies are in terms of color.

After obtaining the color features for the intestinal mucosa, the same routine was applied to the folders containing residues and lumen image samples, allowing the color features for residues and lumen to be obtained.

The color nuances found in artifacts are highly specific, often appearing as extremely white, orange, or green. We identified many artifact regions by detecting colors with very high luminance values—specifically, those with an L coefficient between 253 and 255, regardless of the a and b components. Such color nuances are uncommon in natural photography but can appear in the context of colonoscopy due to specific camera conditions—such as close proximity to the intestinal wall, reflections from the wet mucosa, motion blur from rapid camera movements, underexposed or overexposed frames, and text or message overlays on the video footage. We considered all pixels with an ***L*** (luminance) coefficient above 252 as artifacts; therefore, all very bright nuances were classified as artifacts. For the lumen, after creating the color features from the collected image samples, we also applied a rule to include all the pixels with an ***L*** coefficient less than 50 in the lumen regions, as lumen regions are typically very dark in colonoscopy frames. However, some regions might be dark due to folds in the intestinal mucosa, making it possible to misinterpret them as lumen.

### 2.4. The Database of Colonoscopy Frames

**Figure 4:**
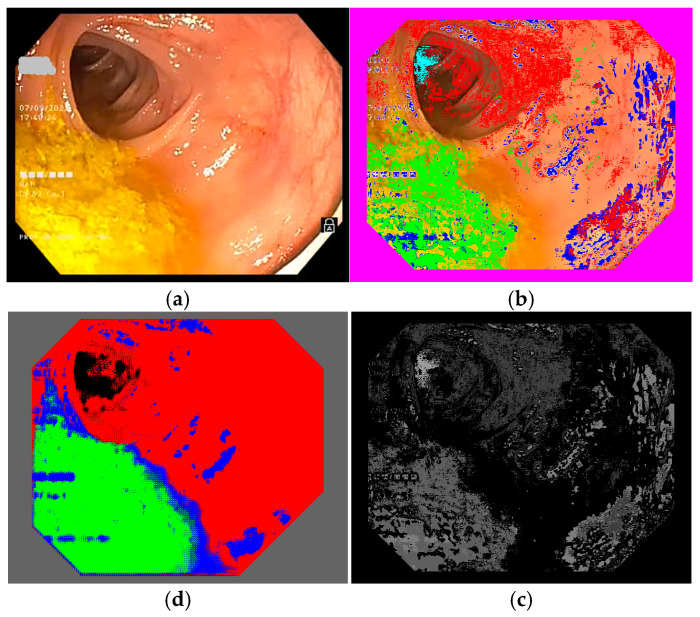
Example of a colonoscopy frame in the training database: (**a**) original frame; (**b**) annotated frame with color features; (**c**) annotated frame in PNG format ready for use in training; (**d**) trained semantic segmentation network applied to the original frame. We used red to mark intestinal mucosa pixels, green for residues, blue for artifacts and cyan for lumen.

**Figure 5:**
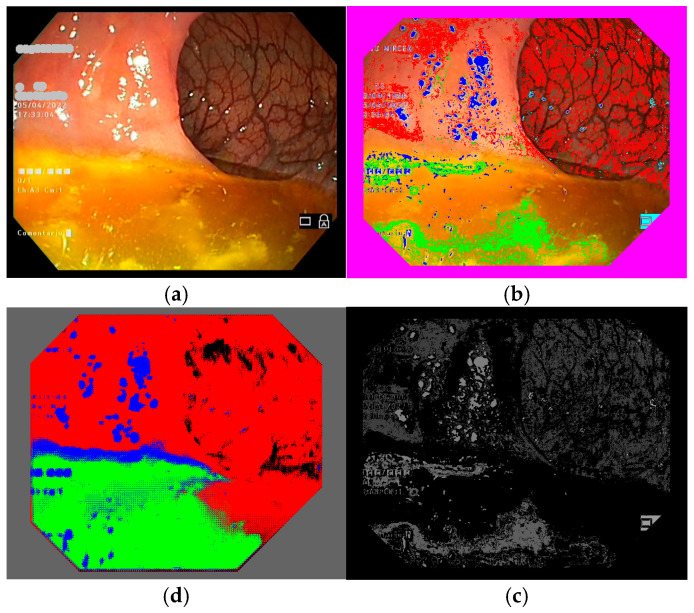
Another example of a colonoscopy frame in the training database. (**a**–**d**) Explanation is the same as Figure 4.

To select the representative frames used to train the semantic segmentation network, we created a database of frames based on 10 colonoscopy videos available in .mp4 format. For each colonoscopy, we created a folder containing the corresponding video file and a subfolder for its extracted frames. The number of frames depended on the duration of the colonoscopy video file and differed from one colonoscopy to another. We developed a routine to extract the frames from a colonoscopy video file and to save them as image files (RGB TIFF, 24 bits/pixels) with a name containing the number of the colonoscopy and the number of each frame (e.g., 04_07563.tiff is the 7563rd frame in colonoscopy video #4). This database was organized into 10 subfolders containing 109,163 TIFF frame files (such as those shown in Figure 4 and Figure 5a). Notably, we ignored 10,136 of the available frames as they were obtained with active narrow band imaging (NBI) filtering.

### 2.5. Classifying Colonoscopy Frame Pixels Based on Region Color Features

To train the semantic segmentation network, annotated colonoscopy frames were needed; that is, frames with pixels marked as belonging to one of the four regions we were interested in. To annotate the frames, we marked intestinal mucosa pixels with pure red (RGB 255 0 0), residues with pure green (RGB 0 255 0), artifacts with pure blue (RGB 0 0 255), and lumen with pure black (RGB 0 0 0).

Colonoscopy frames can be manually annotated by outlining different zones within the frame and assigning them to one of the four regions of interest; however, given that hundreds or even thousands of annotated frames are required, this process can become highly time-consuming, labor-intensive, and prone to errors. To address this challenge, we opted to use our color feature method to identify pixels belonging to the regions of interest, thus automatically creating the annotation files. We implemented a routine to read color features, obtained as detailed above (see Section 2.3), for each colonoscopy frame in TIFF format, producing annotated versions of the frames. It is important to use TIFF for the annotated frames, in order to preserve the colors used to mark regions (see Figure 4 and Figure 5b). If an annotated frame was saved in JPEG format, the pure colors marking annotated pixels would be altered in the compression process. The downside of using TIFF image files is that they occupy more space on hard disks and, so, a computer with higher storage capacity is needed. We ran our routine on all 99,027 frames in our database, creating another 10 sub-folders with annotated images, which had the same file names as the original colonoscopy frame images.

### 2.6. Selecting Annotated Frames for Semantic Segmentation Neural Network Training

As classifying pixels with the color features method described above is not perfect—for instance, because we did not collect sufficient fragments such that all the color nuances of a region (e.g., the intestinal mucosa) could be taken into consideration—we needed to include an intermediary step in which we manually selected the best annotation frames to constitute the training database. We mainly obtained clouds of pixels that belonged to one of the four regions. We expected the neural network to extend the color nuances pertaining to each region and produce a compact segmentation.

Again, we implemented a routine that presents pairs of frames on a screen, namely, an original frame and its annotated version. As we had so many frames to parse (99,027), we decided to browse one frame in each ten available. This was satisfactory, as ten frames covered 40% of a second (colonoscopy video files are saved at a rate of 25 frames per second), and there are not many changes in a colonoscopy over the course of a second. Basically, we selected those annotated frames where residues or artifacts were well-detected (these types of regions are more difficult to automatically classify and are less frequent than the intestinal mucosa). In this step, we were able to evaluate how well the frames had been automatically annotated. At times, we were not satisfied with the annotation results and, so, we went back and changed some parameters or added more image samples, then ran the annotation again. In our final trial, we ended up with 486 original frames (see Figure 4 and Figure 5a) in one folder and 486 annotated frames (see Figure 4 and Figure 5b) in another folder, both in TIFF format and with the same file names in both folders.

### 2.7. Final Training Database for Semantic Segmentation

Our annotation files were obtained in TIFF format, presenting the color code for each of the four regions we were interested in. This helped us to evaluate the quality of annotations directly on the screen of a computer. However, for the sake of training a neural network for semantic segmentation, the annotation files must be grayscale images, with only one color plane (not three, as we have in a color image) and the marking color transformed into a specific gray level (ranging from 1 to 255), while the background is set as black (0).

In Matlab, the annotation files must be presented in PNG format (which occupy minimal space on the disk) and we had to mark the annotation pixels in the following way: intestinal mucosa pixels are transformed from pure red (RGB 255 0 0) to gray level 64, residues pixels are transformed from pure green (RGB 0 255 0) to gray level 94, artifacts pixels are transformed from pure blue (RGB 0 0 255) to gray level 124, and lumen pixels are transformed from pure black (RGB 0 0 0) to gray level 154. We chose these values to allow us to be able to still see the content of the annotation files and to be able to certify that the regions are annotated similarly to the color annotation files (see Figure 4 and Figure 5c). In the end, we obtained two folders—one with the original TIFF frame files and one with the processed PNG annotation files—which were ready to be used to train the semantic segmentation network.

### 2.8. Semantic Segmentation Neural Network Architecture

We were interested in testing whether and how our DL method produced better results than classic image processing approaches. Therefore, we intentionally chose to work with a simple semantic segmentation network from Matlab. Its input layer consisted of 576 × 720 × 3 neurons (i.e., the dimensions of colonoscopy frames in our training database). We used no data augmentation and applied normalization with “zerocenter” in Matlab. The details of the layers of the neural network architecture are provided in Appendix A.

Our semantic segmentation network takes a colonoscopy frame with three RGB color planes as an input, reduces its initial dimension twice through a convolution process, and then reconstructs the initial image as a one-color plane (i.e., as a gray level image) as an output, with the pixel values representing their categorization into one of the four considered regions (Matlab categorical data). In the training process, the neural network was presented successively with all the colonoscopy frames in the training database, and, in each case, the output was compared with the corresponding annotated frame. If differences were reported, the neural network weights were changed such that, the next time it was compared, the differences would be smaller.

### 2.9. Semantic Segmentation Neural Network Training

We performed several training rounds, using different training databases, until we arrived at a better solution for the 486 original colonoscopy frames and their corresponding 486 annotation files. We also tried training with a varying number of epochs. We started with 100 epochs, changing the order of the input frames from one epoch to another, using an initial mini-batch of 25 colonoscopy frames and keeping the learning rate constant at 0.001. The other training tests are detailed in Appendix B. These tests revealed that there were no evident benefits when we trained the network for a large number of epochs (e.g., 400 epochs) at a constant learning rate, nor with a decreasing learning rate. However, we took the last variant (50 epochs with a learning rate of 0.001, 50 epochs with 0.0007, and the last 50 epochs with 0.00049) as a compromise, and used the corresponding model in further tests.

As we rigorously selected the training frames, the neural network was trained sufficiently well with a mini-batch of 48 frames and achieved, in approximatively 6 h, a mini-batch accuracy of 91.48% and a mini-batch loss of 0.0577 (see also Appendix B).

### 2.10. Extracting Statistical Data from Video Colonoscopy Files

With the semantic segmentation network trained to detect pixels pertaining to the four regions of interest (i.e., the intestinal mucosa, residues, artifacts, and lumen), we implemented routines to extract information about how these regions occupy a colonoscopy frame and sum them over an entire video colonoscopy file. This was straightforward to implement, as the output of the neural network provides a category for each pixel in an input colonoscopy frame. We implemented a routine that processes individual colonoscopy frames in TIFF format, allowing us to compare the outcomes of applying the neural network with those achieved using the color feature method. Another routine was developed to process the colonoscopy video files, generating statistics for each region across the entire procedure and producing a video that visually illustrates how the four regions were identified.

## 3. Results

We applied our deep learning model for video colonoscopy file processing to the 10 colonoscopy videos that we initially used to find the colonoscopy frames to train our semantic segmentation network. The results are provided in Table 1, which indicate that the neural network performs much better than the color features method in terms of classifying the pixels into the four considered regions. We then applied it to another set of 7 (different) colonoscopies for testing, and the results are provided in Table 2.

### 3.1. Deep Learning Improvements over the Classical Color Features Method

To find color nuances that pertain to the four regions of interest, we ran a routine that applies the trained neural network to the original TIFF frames that had been extracted from the first set of 10 colonoscopies, as shown in Figure 4 and Figure 5, position (d). This resulted in another folder containing TIFF frames processed using our trained neural network. Then, we applied a routine that extracts the percentages of intestinal mucosa, residues, artifacts, and lumen pixels in every frame and computes these percentages overall for a colonoscopy video. This routine was applied for color features automatic annotation (CFAA) files, with results given in red in Table 1, corresponding to images such as those shown in Figure 4 and Figure 5, position (b), as well as for the deep learning semantic segmentation (DLSS) files, with results given in green in the table, corresponding to images such as those in Figure 4 and Figure 5, position (d).

The comparison between CFAA and DLSS highlights significant differences in terms of their performance. On average, DLSS identified intestinal mucosa in 71.27 ± 13.57% of frames, whereas CFAA only detected 28.75 ± 11.94%, demonstrating a substantial improvement in tissue recognition. Similarly, DLSS showed a higher detection rate for residues (12.30 ± 8.56%) compared to CFAA (3.65 ± 2.46%), indicating increased sensitivity to impurities. In terms of artifacts, DLSS also reported a higher classification rate (8.23 ± 4.37%) than CFAA (4.16 ± 1.06%), suggesting more refined feature extraction but also potential for increased false positives. Regarding lumen detection, DLSS identified a slightly higher percentage (7.38 ± 2.37%) than CFAA (5.21 ± 1.75%), supporting its superior segmentation capabilities. A major advantage of DLSS was its ability to classify all pixels in frames, effectively reducing the unclassified pixels rate from 57.67 ± 11.56% (CFAA) to 0%, showcasing its robustness in terms of image interpretation. Overall, these findings indicate that DLSS provides a more comprehensive and accurate segmentation of colonoscopy images, significantly outperforming CFAA in mucosal tissue recognition and frame classification. However, the increased detection of residues and artifacts suggests the need for further clinical validation, in order to ensure its reliability and optimal quality assessment.

### 3.2. Extracting Pixel Statistics from Video Colonoscopies

A special routine was designed, which takes as input a video colonoscopy file (in .mp4 format), reads its frames sequentially, computes pixel statistics for each frame, and accumulates them to obtain the overall pixel statistics for that colonoscopy. As endoscopists evaluate the level of preparedness for colonoscopy using the Boston scale, which implies determining scores for each of the three segments of the colon, we computed the pixel statistics for the entire colonoscopy and for each of the three segments. As we have no automatic method to detect the passage from one segment to the other, we used the observations of medical evaluators, in terms of the times (minute, second) in the video file where the segments start and end. This was then translated (based on the rate of 25 frames/s) into specific frame numbers for passing from segment 1 to segment 2 and from segment 2 to segment 3.

One aspect that we found important is the effect of artifacts on the pixel statistics for a colonoscopy frame. As the trained neural network was not very good at detecting artifacts, if a frame contains artifacts, it could have a negative influence not only on the readings of pixels belonging to residues (i.e., counting false residues), but also those belonging to the intestinal mucosa or lumen. We extracted pixel statistics in four scenarios:

1. All the frames were taken into consideration;
2. Only the frames with less than 25% artifacts were taken into consideration;
3. Only the frames with less than 40% artifacts were taken into consideration;
4. Only the frames with less than 34% artifacts were taken into consideration.

For each of these scenarios, we applied the trained neural network to the test colonoscopies and analyzed the resulting pixel statistics. We found that there were too many excluded frames in the second scenario while, in the third scenario, there were too few excluded frames. Thus, we opted for the fourth scenario, which provided a good balance between retained and excluded frames. The detailed statistics computed for all seven colonoscopies are provided in the Supplementary S1: All pixel statistics computed for the seven colonoscopies.

Pixel statistics were extracted from test colonoscopies, which were distinct from those used to train the semantic segmentation network. The same videos were then independently assessed by two clinical experts (separate from the performing endoscopists), in order to evaluate the associated bowel preparation quality using the BBPS. The results are summarized in Table 2 (in the left part). In order to take advantage of the percentages computed by our method for the intestinal mucosa, residues, artifacts, and lumen, considered as objective observations, we developed a segment-based evaluation index similar to the BBPS. We concluded that a score of 3 corresponds to a mucosal visualization rate exceeding 70% and a residue visualization rate below 10%, a score of 1 reflects a mucosal visualization rate below 50% and a residue visualization rate exceeding 15%, and a score of 2 corresponds to intermediate values between these thresholds. The results are summarized in the right part of Table 2, representing an evaluation of the overall quality of the colonoscopies, not only an evaluation of bowel preparation.

### 3.3. Results After Applying the Semantic Segmentation Neural Network to Video Colonoscopies

We developed a semantic segmentation network which is capable of estimating the percentages of clearly visible intestinal mucosa, lumen, residues, and artifacts in a colonoscopy frame quickly and with reasonable accuracy. While processes for automatically annotating colonoscopy frames are typically slow (and, thus, time-consuming), our trained neural network is very fast and may even allow for the extraction of pixel statistics in real-time. Through analyzing every frame in a video colonoscopy file, we can identify additional indicators. For example, a high number of frames containing more than 50% residues might suggest inadequate patient preparation for the procedure. Conversely, a high number of frames with more than 75% clearly visible intestinal mucosa could indicate a well-performed colonoscopy examination.

As this is a proof-of-concept study, we applied the trained neural network to seven video colonoscopy files, which had been prepared as previously described, and selected two of them for further discussion. In particular, test colonoscopy T1 received a low Boston score from the physicians, while the other—test colonoscopy T4—received a high Boston score. The main pixel statistics of these colonoscopies are shown comparatively in Table 3.

From the perspective of Boston scores, test colonoscopy T1 showed more detected residues (11% vs. 8%) and frames with over 50% residues (538 vs. 268); however, it had fewer valid frames (6548 vs. 8997). A frame was considered valid if the proportion of artifacts was less than 34%. Segment 1 of test colonoscopy T1 performed poorly, with 21% residues and 24% artifacts. Frames with high residue levels can be automatically saved as evidence of inadequate preparation. In contrast, test colonoscopy T4 had a higher percentage of visible intestinal mucosa (68% vs. 40%) and more valid frames (8997 vs. 6548), despite fewer total frames (10,417 vs. 12,088). It maintained a lower lumen percentage (9% vs. 28%) and had more frames showing over 75% visible mucosa (4371 vs. 453).

Segment analysis showed that segments #1 and #2 of test colonoscopy T1 were the poorest, while segment #3 was comparable to segment #3 in T4. In test colonoscopy T4, segments #2 and #3 performed the best, while segment #1 was less satisfactory. Based on the pixel statistics, overall segment scores based on our model’s pixel statistics were assigned as follows:

- Test colonoscopy T1: 1 point for segments #1 and #2; 2 points for segment #3.
- Test colonoscopy T4: 2 points for segment #1; 3 points each for segments #2 and #3.

We performed Spearman correlation and Cohen’s Kappa agreement analyses on the obtained data to assess the relationships between the expert evaluations using BBPS and the model’s ability to evaluate specific regions of the colon. The Spearman correlation results were as follows: BBPS vs. pixel scores: 0.69; BBPS vs. mucosa pixel percentage: 0.63; BBPS vs. residue pixel percentage: −0.47; BBPS vs. artifact pixel percentage: −0.65. Meanwhile, the agreement analysis using Cohen’s Kappa yielded a value of 0.28. A detailed discussion of these results is presented in the following section.

Table 2 summarizes the pixel statistics for seven test colonoscopies using our trained neural network. Segment scores were based on the detection of the intestinal mucosa, residue, and lumen, excluding artifacts (>34%). Score discrepancies with respect to the BBPS, highlighted in red, were within ±1 point. Again, it should be noted that the BBPS is only for bowel preparation, while the score obtained with our neural network method allows for overall evaluation of a colonoscopy, including bowel preparation. These promising results require extensive testing, in order to confirm the method’s reliability for evaluating video colonoscopies.

## 4. Discussion

In the 21st century, colon-related health issues continue to represent a significant public health concern, leading to considerable morbidity, mortality, and financial burden [22,23]. Among these, colorectal cancer stands out as the third most-prevalent cancer globally, accounting for 9.6% of all cancers worldwide [24]. Colorectal cancer screening, alongside the removal of pre-malignant lesions such as polyps, remains one of the most effective strategies to combat this issue. Similarly, monitoring mucosal healing in the context of inflammatory bowel diseases through colonoscopy has become an accepted approach [25]. However, both technological and human limitations pose challenges to achieving optimal outcomes. With advancements in technology and the emergence of AI systems, these barriers can potentially be overcome. In medicine, including gastroenterology, AI-based systems have emerged as transformative tools allowing for improved healthcare delivery [26]. These systems could be pivotal in overcoming various challenges, serving as essential tools for diagnosis, disease monitoring, and therapeutic decision making, ultimately enhancing patient outcomes.

Most AI-related research in the context of colonoscopy has focused on the detection and characterization of colonic lesions. Many of these studies have demonstrated the positive impacts of AI on lesion detection—notably through improvements in the adenoma detection rate (ADR) [27]—and the assessment of mucosal healing in inflammatory bowel diseases [28]. Despite these advancements, few studies have targeted the objective and automated monitoring of quality indicators in colonoscopy [29,30]. This aspect of the procedure is crucial, as a lack of attention to quality assurance can compromise the reliability and accuracy of medical diagnoses relating to colonoscopy.

### 4.1. Main Results

Our study demonstrates the potential of a semantic segmentation-based deep learning system to improve the evaluation of colonoscopy procedures through accurately identifying four key regions: the intestinal mucosa, residues, artifacts, and lumen. These findings are significant for enhancing the precision of assessments regarding bowel preparation and overall examination quality.

The comparative analysis between our semantic segmentation method and the classical color features method highlighted several critical improvements. The neural network effectively eliminated the issue of unclassified pixels, redistributing them into relevant categories. This capability underscores the strength of DL approaches in learning subtle color nuances associated with the intestinal mucosa and other regions. The adoption of deep learning for semantic segmentation in medical imaging has also been proven to be successful in analyzing complex datasets, especially those collected in noisy or variable environments [31,32]. These studies provided valuable insights into the design of our network, particularly in regions where manual feature-based methods often fail. The accurate detection of residues is particularly noteworthy, as it directly impacts the assessment of bowel preparation quality—a crucial factor for effective colonoscopy. Moreover, the improved identification of artifacts and lumen enhanced our ability to evaluate the technical quality of the procedure, particularly with respect to handling of the colonoscope by the physician.

### 4.2. Comparison with Previous Studies

In their work, Jie Zhou et al. [33] introduced the ENDOANGEL system, a deep convolutional neural network designed for colonoscopy analysis. The model was trained on over 5000 high-quality artifact-free colonoscopy frames, each annotated with the BBPS ground truth by five expert endoscopists. During testing, the system analyzed 30 s colonoscopy video clips, evaluating each frame and assigning the lowest BBPS score within the clip as the final result. Another approach involved providing a BBPS score for every sequence of 10 consecutive colonoscopy frames. In contrast, our neural network was specifically trained to detect artifacts and exclude uninformative colonoscopy frames (i.e., when artifacts covered more than 34% of the image). Additionally, rather than replicating expert-assigned BBPS scores, our approach focused on identifying regions of interest. Through analyzing the percentages of these regions, our system generates an equivalent score to assess the overall quality of a colonoscopy.

Another study, conducted by Cold KM et al. [34], introduced a system for evaluating the quality of fold examination in colonoscopy. This was achieved by training two deep learning neural networks: one designed to exclude non-informative frames and another to assess the visibility of the intestinal walls through detecting the position of the lumen within each frame. Similarly, our approach also considers the lumen region as a key factor in assessing the overall quality of colonoscopies.

In another study, Liu W. et al. [35] aimed to automatically assign a BBPS score to individual colonoscopy frames or entire colonoscopy videos. Their system was trained on 50,000 colonoscopy frames, with annotation files generated automatically by detecting intestinal mucosa and residue regions using fixed hue thresholds in the HSV color system. In our system, we used the Lab color system and avoided setting fixed thresholds, allowing the automated color feature extraction procedure to handle this process. Additionally, while the approach of Liu et al. [35] evaluates the quality of colonoscopy frames based solely on the ratio of residue to intestinal mucosa, producing a BBPS score between 0 and 1, our system incorporates multiple statistical parameters. We analyze four distinct regions—namely, the intestinal mucosa, residues, lumen, and artifacts—to provide a more comprehensive assessment of colonoscopy frame quality.

Our approach to extracting pixel statistics for video colonoscopies allowed us to analyze both global and segment-specific data. Through the use of a threshold for artifact exclusion (34% artifacts per frame), we achieved a balanced trade-off between retaining useful data and minimizing noise. We submitted to our result data to Spearman correlation and Cohen’s Kappa agreement analyses. The Spearman correlation for BBPS vs. the overall pixel score was 0.69; this is a strong positive correlation, indicating that the model-derived pixel scores aligned well with the expert BBPS scores. The coefficient for BBPS vs. the mucosa pixel percentage was 0.63, another positive correlation which indicates that better mucosal visualization by the model (higher percentages) tends to match higher BBPS scores. The correlation for BBPS vs. residues pixel percentages was negative, with a magnitude of 0.47. This reflects a moderate negative correlation, suggesting that higher residue levels correspond to lower BBPS scores, as expected. The BBPS vs. artifact pixel percentage correlation was negative with a magnitude of 0.65. This reflects a strong negative correlation, indicating that frames with more artifacts tend to relate to lower BBPS scores. Furthermore, Cohen’s Kappa agreement analysis yielded a value of 0.28. This represents fair agreement between the BBPS scores and the model’s pixel scores. While not very high, the result is reasonable given the complexity of the task. The correlation between the pixel statistics-based scores and BBPS scores indicates that our system can serve as an objective, automated tool for assessing both bowel preparation quality and overall colonoscopy quality. A summary of the prior studies in comparison to our proposed approach is provided in Table 4.

The analysis of test colonoscopies #T1 and #T4 further validated the utility of our system. The stark contrast in pixel statistics between these two cases illustrates the system’s ability to differentiate between low- and high-quality examinations. Test colonoscopy #T1, characterized by higher residue percentages and fewer valid frames, indicated inadequate bowel preparation and examination quality. Conversely, test colonoscopy #T4 demonstrated superior metrics, with a higher percentage of visible mucosa and more frames meeting the artifact threshold, aligning well with higher BBPS scores.

The segment-wise analysis also provided valuable insights. The identification of segments with sub-optimal or superior quality enables targeted feedback for improving procedural techniques. For instance, in test colonoscopy #T1, segments #1 and #2 were identified as particularly poor, while segment #3 showed relatively better results. In contrast, test colonoscopy #T4 excelled in segments #2 and #3, with segment #1 being slightly less satisfactory. Such detailed assessments can guide physicians in optimizing their techniques and identifying areas for improvement.

### 4.3. Limitations

While our results are promising, the study is limited by the relatively small dataset consisting of 17 colonoscopy videos (10 for training and 7 for testing). The use of a larger dataset is essential, in order to validate and refine the system’s accuracy and reliability. Additionally, our reliance on manual annotations for segment transitions in video colonoscopies introduced potential variability. This challenge is in line with previous AI-based research in endoscopy, where the absence of large, annotated datasets continues to hinder the development of robust and generalizable models [36]. Efforts to integrate multiple independent datasets could provide a solution to overcome this limitation [37]. Future work should focus on developing an automated semantic segmentation algorithm to further address this limitation.

Another limitation is the neural network’s performance in terms of detecting artifacts. There have been various studies focused on leveraging AI for the detection of image artifacts, particularly in the context of gastrointestinal endoscopy. One such study developed a specialized AI algorithm designed to enhance the detection of artifacts in gastrointestinal images, with the goal of quantitatively evaluating the quality of endoscopic visualization [38]. The researchers compared their model to conventional neural networks typically used for image analysis and found that their algorithm significantly outperformed the standard models, achieving higher accuracy in identifying and classifying artifacts [38]. Such an improvement in artifact detection is crucial, as it allows for a more precise assessment of image quality, ultimately leading to better diagnostic outcomes. The ability to accurately distinguish between useful image data and artifacts enhances the reliability of subsequent analyses, helping clinicians to make more informed decisions during medical procedures [38,39]. Frames with significant artifacts can skew pixel statistics, especially for the residues and mucosa. Enhancing the network’s ability to identify artifacts more accurately will be a priority in subsequent research; for instance, we will try to provide a special method to detect each type of artifact, such as bright white spots caused by reflections, which will be handled by a dedicated routine, and error messages overprinted in blue from the colonoscope, which will be treated using another routine. This approach is expected to enhance the precision of artifact detection.

We must also acknowledge the possibility of error sources in training the neural network. The model’s pixel detection does not achieve absolute precision but, rather, provides a reasonable approximation. While this may lead to occasional misclassification of pixels into the four designated regions, the data obtained and evaluated by experts indicated that the proposed AI system was able to generate a reasonably accurate distribution that reflects the real-world situation (with the four regions also being objectively validated by experts). Another potential source of error lies in the annotation process when selecting color features. As our system relies on correctly identifying and classifying each pixel into its corresponding region, the unintended inclusion of color features containing pixels from different regions could lead to training errors. In our case, the selection of color features was conducted meticulously, undergoing multiple verification processes including an IT expert and two gastroenterology specialists.

Detecting knowledge through visual information includes the processes of object detection and localization (i.e., segmentation through bounding boxes or contours), and semantic segmentation. In our approach, we successfully applied semantic segmentation, enabling pixel-level selection and labeling through the use of color nuances and deep learning techniques. It is important to highlight that semantic segmentation assigns class labels to all the pixels in an image. Our method excels at providing detailed information about sparse or loosely defined regions of interest, such as debris scattered on the colon membrane. In contrast, traditional segmentation focuses on well-defined shapes and structures, such as polyps, which are typically used for diagnostic purposes. This requires different tools and is a distinct task, as it deals with specific, well-known anatomical forms [1,2,40]. Semantic segmentation, in contrast, is more flexible and adaptable, enabling the detection and quantification of scattered entities, ultimately contributing to the objective evaluation of colon and bowel preparation quality.

## 5. Conclusions

Our semantic segmentation-based AI system serves as an initial proof-of-concept, demonstrating the feasibility of automated evaluation in the context of colonoscopy quality assessment. In particular, the proposed system may surpass the Boston Bowel Preparation Scale in evaluating colonoscopy quality. Unlike the Boston scale—which assesses only the amount of residual content—our model analyzes and quantifies the proportions of colonic mucosa, residues, and artifacts, offering a more detailed and objective assessment. While this approach shows promise in providing objective and reproducible metrics, its applicability is limited by the scope of the dataset and the need for further development in artifact detection at present. Therefore, future research involving expanding and diversifying the utilized datasets, refining the algorithm’s performance, and validating an automated scoring system with is comparable to the BBPS is essential. These advancements are necessary in order to effectively evaluate the system’s reliability and clinical applicability. With further validation, this technology could support standardized bowel preparation assessments and procedural performance, potentially enhancing patient outcomes.

## Figures and Tables

**Table 1:** Comparison between color features automatic annotation (CFAA, red values) and deep learning semantic segmentation (DLSS, green values) results for the first set of 10 colonoscopies (99,027 frames) used for training.

Colonoscopy	1		2		3		4		5	
	CFAA	DLSS	CFAA	DLSS	CFAA	DLSS	CFAA	DLSS	CFAA	DLSS
#Total Frames	9711	9711	11,981	11,980	8241	8241	6397	6397	20,406	20,406
% Intestinal Mucosa	30.61%	67.65%	45.03%	84.03%	17.25%	48.31%	15.00%	54.40%	44.62%	83.36%
% Residues	6.90%	18.62%	0.64%	2.14%	4.43%	18.80%	7.81%	27.12%	1.56%	3.55%
% Artifacts	3.61%	8.28%	5.59%	4.86%	4.82%	17.65%	4.39%	11.20%	5.63%	5.06%
% Lumen	4.07%	5.45%	8.16%	8.96%	7.29%	15.24%	3.32%	7.28%	7.05%	8.02%
% Not classified	54.81%	0%	40.58%	0%	66.20%	0%	69.49%	0%	41.15%	0%
Colonoscopy	6		7		8		9		10	
	CFAA	DLSS	CFAA	DLSS	CFAA	DLSS	CFAA	DLSS	CFAA	DLSS
#Total Frames	10,394	10,394	13,464	13,464	5373	5373	12,833	12,833	13,129	13,129
% Intestinal Mucosa	44.39%	91.15%	21.61%	79.23%	21.64%	65.42%	28.38%	73.09%	19.00%	66.06%
% Residues	0.52%	0.88%	2.74%	9.17%	3.20%	13.75%	3.71%	13.22%	4.97%	17.94%
% Artifacts	5.34%	3.02%	3.70%	6.92%	4.94%	9.70%	3.16%	7.35%	3.06%	8.27%
% Lumen	5.14%	4.95%	3.63%	4.68%	6.09%	11.13%	3.43%	6.34%	3.96%	7.72%
% Not classified	44.61%	0%	68.33%	0%	64.13%	0%	61.32%	0%	69.01%	0%

**Table 2:** Summarized key pixel statistics for the test colonoscopies and given scores. We marked with red the situations where our method scores differ from the corresponding ones given by endoscopists.

Test Colonoscopy	Boston Scores Given by Endoscopists			Scores Given by Pixel Statistics Analysis			Frames with Less than 34% Artifacts											
							Intestinal Mucosa Pixel Statistics			Residues Pixel Statistics			Artifacts Pixels Statistics			Lumen Pixel Statistics		
T1	1	1	1	1	1	2	34%	34%	61%	21%	13%	2%	24%	21%	20%	21%	33%	17%
	2	1	2															
T2	1	2	2	2	2	**3**	58%	65%	78%	14%	11%	4%	20%	18%	13%	8%	5%	4%
	2	1	2															
T3	1	2	3	1	2	3	34%	44%	62%	14%	4%	0%	21%	19%	14%	31%	33%	23%
	1	2	3															
T4	2	2	2	2	3	**3**	50%	70%	70%	15%	5%	9%	20%	15%	13%	15%	10%	8%
	2	3	2															
T5	1	1	1	1	1	**2**	38%	33%	47%	19%	29%	11%	24%	19%	21%	19%	19%	21%
	2	2	1															
T6	2	2	2	**3**	2	**3**	72%	57%	68%	2%	17%	9%	18%	16%	11%	8%	11%	11%
	2	2	1															
T7	2	2	2	**1**	2	**3**	41%	54%	74%	24%	12%	6%	22%	21%	14%	14%	14%	5%
	2	1	1															

**Table 3:** Comparison between extracted pixel statistics for a poor colonoscopy (T1) and a good colonoscopy (T4). Graphical percentages are presented as follows: intestinal mucosa in red, residues in yellow, artifacts in blue, and lumen in black.

Test Colonoscopy T1	Test Colonoscopy T4
Boston segmental scores: Dr. #1—1 1 1 Dr. #2—2 1 2 (only frames containing less than 34% artifacts were considered valid)	Boston segmental scores: Dr. #1—2 2 2 Dr. #2—2 3 2 (only frames containing less than 34% artifacts were considered valid)
**Total frames = 12,088 Valid frames = 6548** Intestinal mucosa = 40% Residues = 11% Artifacts = 21% Lumen = 28% Frames with intestinal mucosa over 75% = 453 Frames with residues over 50% = 538	**Total frames = 10,417 Valid frames = 8997** Intestinal mucosa = 68% Residues = 8% Artifacts = 14% Lumen = 10% Frames with intestinal mucosa over 75% = 4371 Frames with residues over 50% = 268
**Total segment #1 frames = 1649** **Valid segment #1 frames = 661** Intestinal mucosa = 34% Residues = 21% Artifacts = 24% Lumen = 28% Frames with intestinal mucosa over 75% = 39 Frames with residues over 50% = 134	**Total segment #1 frames = 1274** **Valid segment #1 frames = 888** Intestinal mucosa = 50% Residues = 15% Artifacts = 20% Lumen = 15% Frames with intestinal mucosa over 75% = 56 Frames with residues over 50% = 46
**Total segment #2 frames = 7650** **Valid segment #2 frames = 4478** Intestinal mucosa = 34% Residues = 13% Artifacts = 21% Lumen = 32% Frames with intestinal mucosa over 75% = 89 Frames with residues over 50% = 393	**Total segment #2 frames = 3525** **Valid segment #2 frames = 2846** Intestinal mucosa = 70% Residues = 5% Artifacts = 15% Lumen = 10% Frames with intestinal mucosa over 75% = 1513 Frames with residues over 50% = 30
**Total segment #3 frames = 2789** **Valid segment #3 frames = 1409** Intestinal mucosa = 61% Residues = 2% Artifacts = 20% Lumen = 17% Frames with intestinal mucosa over 75% = 325 Frames with residues over 50% = 11	**Total segment #3 frames = 5618** **Valid segment #3 frames = 5263** Intestinal mucosa = 70% Residues = 9% Artifacts = 13% Lumen = 8% Frames with intestinal mucosa over 75% = 2802 Frames with residues over 50% = 192

**Table 4:** Comparison of prior studies with our approach.

Study	Dataset Size	Annotation Method	Segmentation Focus	Frame Selection	Quality Assessment Approach	Key Contribution
Zhou et al. (ENDOANGEL) [33]	5000 frames	BBPS scoring by 5 experts	BBPS score assignment	Lowest BBPS score per clip	BBPS score per 10 frames	Focus on average BBPS score every 30 s
Cold et al. [34]	Not specified	Frame selection by DL models	Lumen detection for fold assessment	Excludes non-informative frames	Lumen visibility metric	Focus on fold visibility
Liu et al. [35]	50,000 frames	HSV color thresholding	BBPS score based on residue–mucosa ratio	Fixed thresholds	BBPS from residue–mucosa ratio	Automated BBPS computation based on ratio of residue to intestinal mucosa
Our Approach	109,163 frames (486 strictly selected frames for training)	CIELAB color system	Semantic segmentation for mucosa, residues, lumen, and artifacts	Excludes frames with >34% artifacts	Pixel-based segmentation + statistical analysis	Comprehensive frame analysis (intestinal mucosa, residues, artifacts, and lumen) for colonoscopy quality.

**Figure A1:**
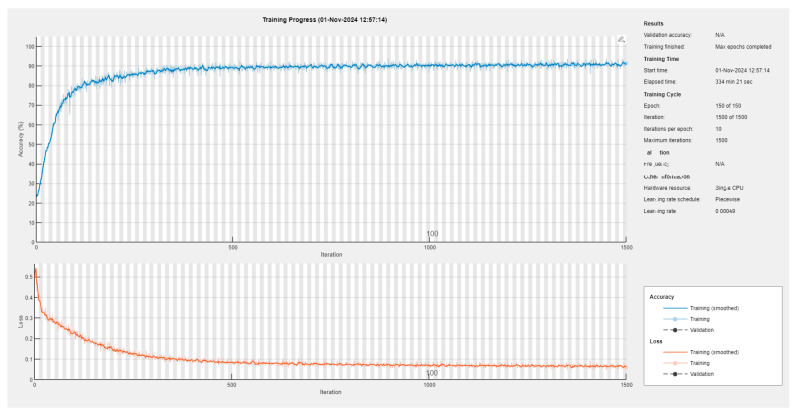
The training session yielding the utilized semantic segmentation network.

## Data Availability

All data necessary for this study are provided within the manuscript and its appendices. The raw video recordings of colonoscopies cannot be shared publicly due to GDPR regulations, as they contain patient-identifiable information. Anonymized versions of these videos may be made available upon reasonable request.

## Supplementary Materials

The following supporting information can be downloaded at: https://www.mdpi.com/article/10.3390/jimaging11030084/s1, Supplementary S1: All pixel statistics computed for the seven colonoscopies.

## Appendix A

**The layers of the neural network architecture:**

There are six down-sampling layers:

1. Two-dimensional convolution 32 3 × 3 convolutions with stride [1 1] and padding [1 1 1 1];
2. ReLU;
3. Two-dimensional max pooling 2 × 2 max pooling with stride [2 2] and padding [0 0 0 0];
4. Two-dimensional convolution 32 3 × 3 convolutions with stride [1 1] and padding [1 1 1 1];
5. ReLU;
6. Two-dimensional max pooling 2 × 2 max pooling with stride [2 2] and padding [0 0 0 0].

The above is followed by four up-sampling layers:

1. Two-dimensionally transposed convolution 32 4 × 4 transposed convolutions with stride [2 2] and cropping [1 1 1 1];
2. ReLU;
3. Two-dimensionally transposed convolution 32 4 × 4 transposed convolutions with stride [2 2] and cropping [1 1 1 1];
4. ReLU.

The final layers assure pixel classification in one of the four regions we were interested in:

1. Two-dimensional convolution 4 1 × 1 convolutions with stride [1 1] and padding [0 0 0 0];
2. Softmax;
3. Pixel-classification-layer cross-entropy loss.

## Appendix B

**Details of training experiments:**

- Training for 50 epochs with a constant learning rate of 0.0003;
- Training for 200 epochs with a constant learning rate of 0.001;
- Training for 400 epochs with a constant learning rate of 0.0002;
- Training for 300 epochs with a decreasing learning rate (100 epochs with 0.001, 100 epochs with 0.0005, and the last 100 epochs with 0.00025);
- Training for 150 epochs with a decreasing learning rate (50 epochs with 0.001, 50 epochs with 0.0007, and the last 50 epochs with 0.00049).

## References

[1] Ahamed M.F., Islam M.R., Nahiduzzaman M., Karim M.J., Ayari M.A., Khandakar A. (2024). Automated Detection of Colorectal Polyp Utilizing Deep Learning Methods With Explainable AI. IEEE Access.

[2] Yue G., Han W., Li S., Zhou T., Lv J., Wang T. (2022). Automated Polyp Segmentation in Colonoscopy Images via Deep Network with Lesion-Aware Feature Selection and Refinement. Biomed. Signal Process Control.

[3] Smeulders A.W.M., Worring M., Santini S., Gupta A., Jain R. (2000). Content-Based Image Retrieval at the End of the Early Years. IEEE Trans. Pattern Anal. Mach. Intell..

[4] Ciobanu A., Luca M., Drug V. Objective Method for Colon Cleansing Evaluation Using Color CIELAB Features. Proceedings of the 2020 International Conference on e-Health and Bioengineering.

[5] Ignat A., Luca M., Ciobanu A. (2016). New Method of Iris Recognition Using Dual Tree Complex Wavelet Transform. Adv. Intell. Syst. Comput..

[6] Ignat A., Luca M. (2016). Rotation Invariant Texture Retrieval Using Dual Tree Complex Wavelet Transform. Proceedings of the Advances in Intelligent Systems and Computing.

[7] Lecun Y., Bengio Y., Hinton G. (2015). Deep Learning. Nature.

[8] Krizhevsky A., Sutskever I., Hinton G.E. (2017). ImageNet Classification with Deep Convolutional Neural Networks. Commun. ACM.

[9] Chen L.C., Papandreou G., Kokkinos I., Murphy K., Yuille A.L. (2016). DeepLab: Semantic Image Segmentation with Deep Convolutional Nets, Atrous Convolution, and Fully Connected CRFs. IEEE Trans. Pattern Anal. Mach. Intell..

[10] Chen L.-C., Papandreou G., Kokkinos I., Murphy K., Yuille A.L. (2014). Semantic Image Segmentation with Deep Convolutional Nets and Fully Connected CRFs. arXiv.

[11] Vaswani A., Shazeer N., Parmar N., Uszkoreit J., Jones L., Gomez A.N., Kaiser Ł., Polosukhin I. (2017). Attention Is All You Need. Adv. Neural Inf. Process Syst..

[12] Hassan C., Piovani D., Spadaccini M., Parigi T., Khalaf K., Facciorusso A., Fugazza A., Rösch T., Bretthauer M., Mori Y. (2023). Variability in Adenoma Detection Rate in Control Groups of Randomized Colonoscopy Trials: A Systematic Review and Meta-Analysis. Gastrointest. Endosc..

[13] Nazarian S., Glover B., Ashrafian H., Darzi A., Teare J. (2021). Diagnostic Accuracy of Artificial Intelligence and Computer-Aided Diagnosis for the Detection and Characterization of Colorectal Polyps: Systematic Review and Meta-Analysis. J. Med. Internet Res..

[14] Hassan C., East J., Radaelli F., Spada C., Benamouzig R., Bisschops R., Bretthauer M., Dekker E., Dinis-Ribeiro M., Ferlitsch M. (2019). Bowel Preparation for Colonoscopy: European Society of Gastrointestinal Endoscopy (Esge) Guideline-Update 2019. Endoscopy.

[15] Kastenberg D., Bertiger G., Brogadir S. (2018). Bowel Preparation Quality Scales for Colonoscopy. World J. Gastroenterol..

[16] Kim J., Choi J.M., Lee J., Han Y.M., Jin E.H., Lim J.H., Bae J.H., Seo J.Y. (2024). Boston Bowel Preparation Scale Score 6 Has More Missed Lesions Compared with 7–9. Sci. Rep..

[17] Saraiva S., Rosa I., Pereira A.D. (2021). Use of the Boston Bowel Preparation Scale in the Real Life Setting: What Affects It?. Rev. Esp. Enfermedades Dig..

[18] Jha D., Ali S., Tomar N.K., Johansen H.D., Johansen D., Rittscher J., Riegler M.A., Halvorsen P. (2021). Real-Time Polyp Detection, Localization and Segmentation in Colonoscopy Using Deep Learning. IEEE Access.

[19] Thakkar S., Carleton N.M., Rao B., Syed A. (2020). Use of Artificial Intelligence-Based Analytics From Live Colonoscopies to Optimize the Quality of the Colonoscopy Examination in Real Time: Proof of Concept. Gastroenterology.

[20] Parasa S., Berzin T., Leggett C., Gross S., Repici A., Ahmad O.F., Chiang A., Coelho-Prabhu N., Cohen J., Dekker E. (2025). Consensus Statements on the Current Landscape of Artificial Intelligence Applications in Endoscopy, Addressing Roadblocks, and Advancing Artificial Intelligence in Gastroenterology. Gastrointest. Endosc..

[21] International Color Consortium Specification ICC.1:2022 (Profile Version 4.4.0.0) Image Technology Color Management—Architecture, Profile Format and Data Structure. https://www.color.org/icc_specs2.xalter.

[22] Klimeck L., Heisser T., Hoffmeister M., Brenner H. (2023). Colorectal Cancer: A Health and Economic Problem. Best. Pract. Res. Clin. Gastroenterol..

[23] (2023). The Economic Burden of Inflammatory Bowel Disease. Lancet Gastroenterol. Hepatol..

[24] BrBray F., Laversanne M., Sung H., Ferlay J., Siegel R.L., Soerjomataram I., Jemal A. (2024). Global Cancer Statistics 2022: GLOBOCAN Estimates of Incidence and Mortality Worldwide for 36 Cancers in 185 Countries. CA Cancer J Clin.

[25] Otte M.L., Tamang R.L., Papapanagiotou J., Ahmad R., Dhawan P., Singh A.B. (2023). Mucosal Healing and Inflammatory Bowel Disease: Therapeutic Implications and New Targets. World J. Gastroenterol..

[26] Abadir A.P., Ali M.F., Karnes W., Samarasena J.B. (2020). Artificial Intelligence in Gastrointestinal Endoscopy. Clin. Endosc..

[27] Hassan C., Spadaccini M., Iannone A., Maselli R., Jovani M., Chandrasekar V.T., Antonelli G., Yu H., Areia M., Dinis-Ribeiro M. (2021). Performance of Artificial Intelligence in Colonoscopy for Adenoma and Polyp Detection: A Systematic Review and Meta-Analysis. Gastrointest. Endosc..

[28] Nakase H., Hirano T., Wagatsuma K., Ichimiya T., Yamakawa T., Yokoyama Y., Hayashi Y., Hirayama D., Kazama T., Yoshii S. (2021). Artificial Intelligence-Assisted Endoscopy Changes the Definition of Mucosal Healing in Ulcerative Colitis. Dig. Endosc..

[29] Zhou W., Yao L., Wu H., Zheng B., Hu S., Zhang L., Li X., He C., Wang Z., Li Y. (2021). Multi-Step Validation of a Deep Learning-Based System for the Quantification of Bowel Preparation: A Prospective, Observational Study. Lancet Digit. Health.

[30] Lee J.Y., Calderwood A.H., Karnes W., Requa J., Jacobson B.C., Wallace M.B. (2022). Artificial Intelligence for the Assessment of Bowel Preparation. Gastrointest. Endosc..

[31] Chu Y., Yang X., Li H., Ai D., Ding Y., Fan J., Song H., Yang J. (2020). Multi-Level Feature Aggregation Network for Instrument Identification of Endoscopic Images. Phys. Med. Biol..

[32] Rayed M.E., Islam S.M.S., Niha S.I., Jim J.R., Kabir M.M., Mridha M.F. (2024). Deep Learning for Medical Image Segmentation: State-of-the-Art Advancements and Challenges. Inform. Med. Unlocked.

[33] Zhou J., Wu L., Wan X., Shen L., Liu J., Zhang J., Jiang X., Wang Z., Yu S., Kang J. (2020). A Novel Artificial Intelligence System for the Assessment of Bowel Preparation (with Video). Gastrointest. Endosc..

[34] Cold K.M., Heen A., Vamadevan A., Vilmann A.S., Konge L., Rasmussen M., Svendsen M.B.S. Development and Validation of the Open-Source Automatic Bowel Preparation Scale. Gastrointest. Endosc..

[35] Liu W., Wu Y., Yuan X., Zhang J., Zhou Y., Zhang W., Zhu P., Tao Z., He L., Hu B. (2022). Artificial Intelligence-Based Assessments of Colonoscopic Withdrawal Technique: A New Method for Measuring and Enhancing the Quality of Fold Examination. Endoscopy.

[36] Taghiakbari M., Mori Y., von Renteln D. (2021). Artificial Intelligence-Assisted Colonoscopy: A Review of Current State of Practice and Research. World J. Gastroenterol..

[37] Van Der Sommen F., De Groof J., Struyvenberg M., Van Der Putten J., Boers T., Fockens K., Schoon E.J., Curvers W., De With P., Mori Y. (2020). Machine Learning in GI Endoscopy: Practical Guidance in How to Interpret a Novel Field. Gut.

[38] Sun W., Li P., Liang Y., Feng Y., Zhao L. (2023). Detection of Image Artifacts Using Improved Cascade Region-Based CNN for Quality Assessment of Endoscopic Images. Bioengineering.

[39] Zhang C., Zhang N., Wang D., Cao Y., Liu B. Artifact Detection in Endoscopic Video with Deep Convolutional Neural Networks. Proceedings of the 2020 2nd International Conference on Transdisciplinary AI, TransAI 2020.

[40] Kvasir-SEG Benchmark (Medical Image Segmentation)|Papers with Code. https://paperswithcode.com/sota/medical-image-segmentation-on-kvasir-seg.

