# Supplementary material for: Deep Learning-Based Semantic Segmentation for Objective Colonoscopy Quality Assessment
Source: J Imaging. 2025 Mar 18;11(3):84. doi: 10.3390/jimaging11030084 (PMC11943454; doi:10.3390/jimaging11030084)
Supplement: Supplementary file 1 [file jimaging-11-00084-s001.zip › jimaging-3495813-supplementary.pdf]

Supplementary S1: All pixel statistics computed for the seven colonoscopies

Test Colonoscopy T1
9N 150 0.001 0.0007 0.0049 (**Trained network #9N, 150 epochs, learning rate for three stages of 50 epochs**)
Artefacts under 34%

Total frames = 12088
Valid frames = 6548

Average Intestinal Mucosa = 39.70 %
Average Residues = 11.13 %
Average Artefacts = 20.99 %
Average Lumen = 28.17 %

Frames with Intestinal Mucosa under 15% = 1179
Frames with Intestinal Mucosa between 15% and 50% = 2919
Frames with Intestinal Mucosa between 50% and 75% = 1997
Frames with Intestinal Mucosa over 75% = 453

Frames with Residues under 5% = 4448
Frames with Residues between 5% and 25% = 943
Frames with Residues between 25% and 50% = 619
Frames with Residues over 50% = 538

Frames with Artefacts under 5% = 200
Frames with Artefacts between 5% and 25% = 3869
Frames with Artefacts between 25% and 34% = 2479
Frames with Artefacts over 34% = 0

Frames with Lumen under 5% = 61
Frames with Lumen between 5% and 25% = 3454
Frames with Lumen between 25% and 50% = 1814
Frames with Lumen over 50% = 1219

Segment #1
Total segment frames = 1649
Valid segment frames = 661

Average Intestinal Mucosa = 34.26 %
Average Residues = 21.20 %
Average Artefacts = 23.59 %
Average Lumen = 20.96 %

Frames with Intestinal Mucosa under 15% = 187
Frames with Intestinal Mucosa between 15% and 50% = 270
Frames with Intestinal Mucosa between 50% and 75% = 165
Frames with Intestinal Mucosa over 75% = 39

Frames with Residues under 5% = 292
Frames with Residues between 5% and 25% = 155
Frames with Residues between 25% and 50% = 80
Frames with Residues over 50% = 134

Frames with Artefacts under 5% = 28
Frames with Artefacts between 5% and 25% = 263
Frames with Artefacts between 25% and 34% = 370
Frames with Artefacts over 34% = 0

Frames with Lumen under 5% = 6
Frames with Lumen between 5% and 25% = 431
Frames with Lumen between 25% and 50% = 135
Frames with Lumen over 50% = 89

Segment #2
Total segment frames = 7650
Valid segment frames = 4478

Average Intestinal Mucosa = 33.73 %
Average Residues = 12.66 %
Average Artefacts = 20.98 %
Average Lumen = 32.63 %

Frames with Intestinal Mucosa under 15% = 935
Frames with Intestinal Mucosa between 15% and 50% = 2443
Frames with Intestinal Mucosa between 50% and 75% = 1011
Frames with Intestinal Mucosa over 75% = 89

Frames with Residues under 5% = 2823
Frames with Residues between 5% and 25% = 733
Frames with Residues between 25% and 50% = 529
Frames with Residues over 50% = 393

Frames with Artefacts under 5% = 155
Frames with Artefacts between 5% and 25% = 2675
Frames with Artefacts between 25% and 34% = 1648
Frames with Artefacts over 34% = 0

Frames with Lumen under 5% = 23

Frames with Lumen between 5% and 25% = 1915
Frames with Lumen between 25% and 50% = 1504
Frames with Lumen over 50% = 1036

Segment #3
Total segment frames = 2789
Valid segment frames = 1409

Average Intestinal Mucosa = 61.24 %
Average Residues = 1.53 %
Average Artefacts = 19.83 %
Average Lumen = 17.40 %

Frames with Intestinal Mucosa under 15% = 57
Frames with Intestinal Mucosa between 15% and 50% = 206
Frames with Intestinal Mucosa between 50% and 75% = 821
Frames with Intestinal Mucosa over 75% = 325

Frames with Residues under 5% = 1333
Frames with Residues between 5% and 25% = 55
Frames with Residues between 25% and 50% = 10
Frames with Residues over 50% = 11

Frames with Artefacts under 5% = 17
Frames with Artefacts between 5% and 25% = 931
Frames with Artefacts between 25% and 34% = 461
Frames with Artefacts over 34% = 0

Frames with Lumen under 5% = 32
Frames with Lumen between 5% and 25% = 1108
Frames with Lumen between 25% and 50% = 175
Frames with Lumen over 50% = 94

Test Colonoscopy T2
9N 150 0.001 0.0007 0.00049
Artefacts under 34%

Total frames = 9787
Valid frames = 8525

Average Intestinal Mucosa = 66.17 %
Average Residues = 10.21 %
Average Artefacts = 17.41 %
Average Lumen = 6.22 %

Frames with Intestinal Mucosa under 15% = 266
Frames with Intestinal Mucosa between 15% and 50% = 1365
Frames with Intestinal Mucosa between 50% and 75% = 3495
Frames with Intestinal Mucosa over 75% = 3399

Frames with Residues under 5% = 5121
Frames with Residues between 5% and 25% = 2187
Frames with Residues between 25% and 50% = 873
Frames with Residues over 50% = 344

Frames with Artefacts under 5% = 516
Frames with Artefacts between 5% and 25% = 6146
Frames with Artefacts between 25% and 34% = 1863
Frames with Artefacts over 34% = 0

Frames with Lumen under 5% = 5768
Frames with Lumen between 5% and 25% = 2232
Frames with Lumen between 25% and 50% = 445
Frames with Lumen over 50% = 80

Segment #1
Total segment frames = 3799
Valid segment frames = 3157

Average Intestinal Mucosa = 57.96 %
Average Residues = 14.24 %
Average Artefacts = 19.54 %
Average Lumen = 8.26 %

Frames with Intestinal Mucosa under 15% = 103
Frames with Intestinal Mucosa between 15% and 50% = 879
Frames with Intestinal Mucosa between 50% and 75% = 1507

Frames with Intestinal Mucosa over 75% = 668

Frames with Residues under 5% = 1552
Frames with Residues between 5% and 25% = 952
Frames with Residues between 25% and 50% = 427
Frames with Residues over 50% = 226

Frames with Artefacts under 5% = 183
Frames with Artefacts between 5% and 25% = 1953
Frames with Artefacts between 25% and 34% = 1021
Frames with Artefacts over 34% = 0

Frames with Lumen under 5% = 1707
Frames with Lumen between 5% and 25% = 1192
Frames with Lumen between 25% and 50% = 227
Frames with Lumen over 50% = 31

Segment #2
Total segment frames = 3525
Valid segment frames = 3083

Average Intestinal Mucosa = 65.44 %
Average Residues = 10.91 %
Average Artefacts = 18.21 %
Average Lumen = 5.43 %

Frames with Intestinal Mucosa under 15% = 137
Frames with Intestinal Mucosa between 15% and 50% = 388
Frames with Intestinal Mucosa between 50% and 75% = 1412
Frames with Intestinal Mucosa over 75% = 1146

Frames with Residues under 5% = 1727
Frames with Residues between 5% and 25% = 869
Frames with Residues between 25% and 50% = 396
Frames with Residues over 50% = 91

Frames with Artefacts under 5% = 88
Frames with Artefacts between 5% and 25% = 2351
Frames with Artefacts between 25% and 34% = 644
Frames with Artefacts over 34% = 0

Frames with Lumen under 5% = 2261
Frames with Lumen between 5% and 25% = 648
Frames with Lumen between 25% and 50% = 145
Frames with Lumen over 50% = 29

Segment #3
Total segment frames = 2463
Valid segment frames = 2285

Average Intestinal Mucosa = 78.49 %
Average Residues = 3.67 %
Average Artefacts = 13.39 %
Average Lumen = 4.45 %
Average percentage not detected = 0.00 %

Frames with Intestinal Mucosa under 15% = 26
Frames with Intestinal Mucosa between 15% and 50% = 98
Frames with Intestinal Mucosa between 50% and 75% = 576
Frames with Intestinal Mucosa over 75% = 1585

Frames with Residues under 5% = 1842
Frames with Residues between 5% and 25% = 366
Frames with Residues between 25% and 50% = 50
Frames with Residues over 50% = 27

Frames with Artefacts under 5% = 245
Frames with Artefacts between 5% and 25% = 1842
Frames with Artefacts between 25% and 34% = 198
Frames with Artefacts over 34% = 0

Frames with Lumen under 5% = 1800
Frames with Lumen between 5% and 25% = 392
Frames with Lumen between 25% and 50% = 73
Frames with Lumen over 50% = 20

Test Colonoscopy T3
9N 150 0.001 0.0007 0.00049
Artefacts under 34%

Total frames = 7535
Valid frames = 4011

Average Intestinal Mucosa = 43.50 %
Average Residues = 7.04 %
Average Artefacts = 18.80 %
Average Lumen = 30.66 %
Average percentage not detected = 0.00 %

Frames with Intestinal Mucosa under 15% = 910
Frames with Intestinal Mucosa between 15% and 50% = 1165
Frames with Intestinal Mucosa between 50% and 75% = 1314
Frames with Intestinal Mucosa over 75% = 622

Frames with Residues under 5% = 3260
Frames with Residues between 5% and 25% = 420
Frames with Residues between 25% and 50% = 99
Frames with Residues over 50% = 232

Frames with Artefacts under 5% = 195
Frames with Artefacts between 5% and 25% = 2650
Frames with Artefacts between 25% and 34% = 1166
Frames with Artefacts over 34% = 0

Frames with Lumen under 5% = 563
Frames with Lumen between 5% and 25% = 1494
Frames with Lumen between 25% and 50% = 902
Frames with Lumen over 50% = 1052

Segment #1
Total segment frames = 3295
Valid segment frames = 1471

Average Intestinal Mucosa = 34.00 %
Average Residues = 13.76 %
Average Artefacts = 20.80 %
Average Lumen = 31.44 %

Frames with Intestinal Mucosa under 15% = 549
Frames with Intestinal Mucosa between 15% and 50% = 305

Frames with Intestinal Mucosa between 50% and 75% = 577
Frames with Intestinal Mucosa over 75% = 40

Frames with Residues under 5% = 1092
Frames with Residues between 5% and 25% = 117
Frames with Residues between 25% and 50% = 67
Frames with Residues over 50% = 195

Frames with Artefacts under 5% = 86
Frames with Artefacts between 5% and 25% = 772
Frames with Artefacts between 25% and 34% = 613
Frames with Artefacts over 34% = 0

Frames with Lumen under 5% = 141
Frames with Lumen between 5% and 25% = 699
Frames with Lumen between 25% and 50% = 169
Frames with Lumen over 50% = 462

Segment #2
Total segment frames = 3455
Valid segment frames = 1837

Average Intestinal Mucosa = 43.86 %
Average Residues = 4.34 %
Average Artefacts = 18.94 %
Average Lumen = 32.86 %

Frames with Intestinal Mucosa under 15% = 305
Frames with Intestinal Mucosa between 15% and 50% = 779
Frames with Intestinal Mucosa between 50% and 75% = 406
Frames with Intestinal Mucosa over 75% = 347

Frames with Residues under 5% = 1465
Frames with Residues between 5% and 25% = 303
Frames with Residues between 25% and 50% = 32
Frames with Residues over 50% = 37

Frames with Artefacts under 5% = 51
Frames with Artefacts between 5% and 25% = 1304
Frames with Artefacts between 25% and 34% = 482
Frames with Artefacts over 34% = 0

Frames with Lumen under 5% = 242
Frames with Lumen between 5% and 25% = 505
Frames with Lumen between 25% and 50% = 603

Frames with Lumen over 50% = 487

Segment #3
Total segment frames = 785
Valid segment frames = 703

Average Intestinal Mucosa = 62.44 %
Average Residues = 0.03 %
Average Artefacts = 14.25 %
Average Lumen = 23.28 %

Frames with Intestinal Mucosa under 15% = 56
Frames with Intestinal Mucosa between 15% and 50% = 81
Frames with Intestinal Mucosa between 50% and 75% = 331
Frames with Intestinal Mucosa over 75% = 235

Frames with Residues under 5% = 703
Frames with Residues between 5% and 25% = 0
Frames with Residues between 25% and 50% = 0
Frames with Residues over 50% = 0

Frames with Artefacts under 5% = 58
Frames with Artefacts between 5% and 25% = 574
Frames with Artefacts between 25% and 34% = 71
Frames with Artefacts over 34% = 0

Frames with Lumen under 5% = 180
Frames with Lumen between 5% and 25% = 290
Frames with Lumen between 25% and 50% = 130
Frames with Lumen over 50% = 103

Test Colonoscopy T4
9N 150 0.001 0.0007 0.00049
Artefacts under 34%

Total frames = 10417
Valid frames = 8997

Average Intestinal Mucosa = 67.97 %
Average Residues = 8.28 %
Average Artefacts = 14.34 %
Average Lumen = 9.42 %

Frames with Intestinal Mucosa under 15% = 336
Frames with Intestinal Mucosa between 15% and 50% = 1586
Frames with Intestinal Mucosa between 50% and 75% = 2704
Frames with Intestinal Mucosa over 75% = 4371

Frames with Residues under 5% = 6285
Frames with Residues between 5% and 25% = 1440
Frames with Residues between 25% and 50% = 1004
Frames with Residues over 50% = 268

Frames with Artefacts under 5% = 1215
Frames with Artefacts between 5% and 25% = 6515
Frames with Artefacts between 25% and 34% = 1267
Frames with Artefacts over 34% = 0

Frames with Lumen under 5% = 5914
Frames with Lumen between 5% and 25% = 2017
Frames with Lumen between 25% and 50% = 567
Frames with Lumen over 50% = 499

Segment #1
Total segment frames = 1274
Valid segment frames = 888

Average Intestinal Mucosa = 50.35 %
Average Residues = 14.67 %
Average Artefacts = 19.61 %
Average Lumen = 15.36 %

Frames with Intestinal Mucosa under 15% = 65
Frames with Intestinal Mucosa between 15% and 50% = 323

Frames with Intestinal Mucosa between 50% and 75% = 444
Frames with Intestinal Mucosa over 75% = 56

Frames with Residues under 5% = 355
Frames with Residues between 5% and 25% = 335
Frames with Residues between 25% and 50% = 152
Frames with Residues over 50% = 46

Frames with Artefacts under 5% = 79
Frames with Artefacts between 5% and 25% = 516
Frames with Artefacts between 25% and 34% = 293
Frames with Artefacts over 34% = 0

Frames with Lumen under 5% = 419
Frames with Lumen between 5% and 25% = 281
Frames with Lumen between 25% and 50% = 108
Frames with Lumen over 50% = 80

Segment #2
Total segment frames = 3525
Valid segment frames = 2846

Average Intestinal Mucosa = 69.68 %
Average Residues = 5.00 %
Average Artefacts = 15.37 %
Average Lumen = 9.95 %

Frames with Intestinal Mucosa under 15% = 88
Frames with Intestinal Mucosa between 15% and 50% = 443
Frames with Intestinal Mucosa between 50% and 75% = 802
Frames with Intestinal Mucosa over 75% = 1513

Frames with Residues under 5% = 2219
Frames with Residues between 5% and 25% = 444
Frames with Residues between 25% and 50% = 153
Frames with Residues over 50% = 30

Frames with Artefacts under 5% = 308
Frames with Artefacts between 5% and 25% = 2007
Frames with Artefacts between 25% and 34% = 531
Frames with Artefacts over 34% = 0

Frames with Lumen under 5% = 2052
Frames with Lumen between 5% and 25% = 385
Frames with Lumen between 25% and 50% = 176

Frames with Lumen over 50% = 233

Segment #3
Total segment frames = 5618
Valid segment frames = 5263

Average Intestinal Mucosa = 70.01 %
Average Residues = 8.98 %
Average Artefacts = 12.89 %
Average Lumen = 8.12 %

Frames with Intestinal Mucosa under 15% = 183
Frames with Intestinal Mucosa between 15% and 50% = 820
Frames with Intestinal Mucosa between 50% and 75% = 1458
Frames with Intestinal Mucosa over 75% = 2802

Frames with Residues under 5% = 3711
Frames with Residues between 5% and 25% = 661
Frames with Residues between 25% and 50% = 699
Frames with Residues over 50% = 192

Frames with Artefacts under 5% = 828
Frames with Artefacts between 5% and 25% = 3992
Frames with Artefacts between 25% and 34% = 443
Frames with Artefacts over 34% = 0

Frames with Lumen under 5% = 3443
Frames with Lumen between 5% and 25% = 1351
Frames with Lumen between 25% and 50% = 283
Frames with Lumen over 50% = 186

Test Colonoscopy T5
9N 150 0.001 0.0007 0.00049
Artefacts under 34%

Total frames = 6647
Valid frames = 3325

Average Intestinal Mucosa = 38.37 %
Average Residues = 22.06 %
Average Artefacts = 19.68 %
Average Lumen = 19.89 %
Average percentage not detected = 0.00 %

Frames with Intestinal Mucosa under 15% = 620
Frames with Intestinal Mucosa between 15% and 50% = 1539
Frames with Intestinal Mucosa between 50% and 75% = 913
Frames with Intestinal Mucosa over 75% = 253

Frames with Residues under 5% = 1358
Frames with Residues between 5% and 25% = 952
Frames with Residues between 25% and 50% = 346
Frames with Residues over 50% = 669

Frames with Artefacts under 5% = 403
Frames with Artefacts between 5% and 25% = 1674
Frames with Artefacts between 25% and 34% = 1248
Frames with Artefacts over 34% = 0

Frames with Lumen under 5% = 1546
Frames with Lumen between 5% and 25% = 658
Frames with Lumen between 25% and 50% = 593
Frames with Lumen over 50% = 528

Segment #1
Total segment frames = 1250
Valid segment frames = 316

Average Intestinal Mucosa = 38.30 %
Average Residues = 18.79 %
Average Artefacts = 23.70 %
Average Lumen = 19.21 %

Frames with Intestinal Mucosa under 15% = 81

Frames with Intestinal Mucosa between 15% and 50% = 101
Frames with Intestinal Mucosa between 50% and 75% = 132
Frames with Intestinal Mucosa over 75% = 2

Frames with Residues under 5% = 144
Frames with Residues between 5% and 25% = 87
Frames with Residues between 25% and 50% = 41
Frames with Residues over 50% = 44

Frames with Artefacts under 5% = 14
Frames with Artefacts between 5% and 25% = 113
Frames with Artefacts between 25% and 34% = 189
Frames with Artefacts over 34% = 0

Frames with Lumen under 5% = 161
Frames with Lumen between 5% and 25% = 66
Frames with Lumen between 25% and 50% = 43
Frames with Lumen over 50% = 46

Segment #2
Total segment frames = 2925
Valid segment frames = 1855

Average Intestinal Mucosa = 33.16 %
Average Residues = 29.49 %
Average Artefacts = 18.16 %
Average Lumen = 19.19 %

Frames with Intestinal Mucosa under 15% = 348
Frames with Intestinal Mucosa between 15% and 50% = 1065
Frames with Intestinal Mucosa between 50% and 75% = 356
Frames with Intestinal Mucosa over 75% = 86

Frames with Residues under 5% = 626
Frames with Residues between 5% and 25% = 479
Frames with Residues between 25% and 50% = 151
Frames with Residues over 50% = 599

Frames with Artefacts under 5% = 354
Frames with Artefacts between 5% and 25% = 884
Frames with Artefacts between 25% and 34% = 617
Frames with Artefacts over 34% = 0

Frames with Lumen under 5% = 906
Frames with Lumen between 5% and 25% = 347

Frames with Lumen between 25% and 50% = 288
Frames with Lumen over 50% = 314

Segment #3
Total segment frames = 2472
Valid segment frames = 1154

Average Intestinal Mucosa = 46.75 %
Average Residues = 11.02 %
Average Artefacts = 21.04 %
Average Lumen = 21.20 %

Frames with Intestinal Mucosa under 15% = 191
Frames with Intestinal Mucosa between 15% and 50% = 373
Frames with Intestinal Mucosa between 50% and 75% = 425
Frames with Intestinal Mucosa over 75% = 165

Frames with Residues under 5% = 588
Frames with Residues between 5% and 25% = 386
Frames with Residues between 25% and 50% = 154
Frames with Residues over 50% = 26

Frames with Artefacts under 5% = 35
Frames with Artefacts between 5% and 25% = 677
Frames with Artefacts between 25% and 34% = 442
Frames with Artefacts over 34% = 0

Frames with Lumen under 5% = 479
Frames with Lumen between 5% and 25% = 245
Frames with Lumen between 25% and 50% = 262
Frames with Lumen over 50% = 168

Test Colonoscopy T6
9N 150 0.001 0.0007 0.00049
Artefacts under 34%

Total frames = 16043
Valid frames = 14342

Average Intestinal Mucosa = 63.17 %
Average Residues = 12.69 %
Average Artefacts = 15.22 %
Average Lumen = 10.76 %
Average percentage not detected = 0.00 %

Frames with Intestinal Mucosa under 15% = 946
Frames with Intestinal Mucosa between 15% and 50% = 3203
Frames with Intestinal Mucosa between 50% and 75% = 4729
Frames with Intestinal Mucosa over 75% = 5464

Frames with Residues under 5% = 9365
Frames with Residues between 5% and 25% = 2046
Frames with Residues between 25% and 50% = 1600
Frames with Residues over 50% = 1331

Frames with Artefacts under 5% = 2129
Frames with Artefacts between 5% and 25% = 10057
Frames with Artefacts between 25% and 34% = 2156
Frames with Artefacts over 34% = 0

Frames with Lumen under 5% = 8292
Frames with Lumen between 5% and 25% = 4176
Frames with Lumen between 25% and 50% = 1171
Frames with Lumen over 50% = 703

Segment #1
Total segment frames = 2375
Valid segment frames = 2160

Average Intestinal Mucosa = 72.07 %
Average Residues = 1.78 %
Average Artefacts = 17.71 %
Average Lumen = 8.44 %

Frames with Intestinal Mucosa under 15% = 8

Frames with Intestinal Mucosa between 15% and 50% = 167
Frames with Intestinal Mucosa between 50% and 75% = 821
Frames with Intestinal Mucosa over 75% = 1164

Frames with Residues under 5% = 1997
Frames with Residues between 5% and 25% = 135
Frames with Residues between 25% and 50% = 28
Frames with Residues over 50% = 0

Frames with Artefacts under 5% = 73
Frames with Artefacts between 5% and 25% = 1682
Frames with Artefacts between 25% and 34% = 405
Frames with Artefacts over 34% = 0

Frames with Lumen under 5% = 1105
Frames with Lumen between 5% and 25% = 890
Frames with Lumen between 25% and 50% = 121
Frames with Lumen over 50% = 44

Segment #2
Total segment frames = 9875
Valid segment frames = 8614

Average Intestinal Mucosa = 56.86 %
Average Residues = 16.53 %
Average Artefacts = 15.84 %
Average Lumen = 10.77 %

Frames with Intestinal Mucosa under 15% = 763
Frames with Intestinal Mucosa between 15% and 50% = 2279
Frames with Intestinal Mucosa between 50% and 75% = 3137
Frames with Intestinal Mucosa over 75% = 2435

Frames with Residues under 5% = 4697
Frames with Residues between 5% and 25% = 1530
Frames with Residues between 25% and 50% = 1336
Frames with Residues over 50% = 1051

Frames with Artefacts under 5% = 988
Frames with Artefacts between 5% and 25% = 6148
Frames with Artefacts between 25% and 34% = 1478
Frames with Artefacts over 34% = 0

Frames with Lumen under 5% = 5188
Frames with Lumen between 5% and 25% = 2261

Frames with Lumen between 25% and 50% = 668
Frames with Lumen over 50% = 497

Segment #3
Total segment frames = 3793
Valid segment frames = 3568

Average Intestinal Mucosa = 68.46 %
Average Residues = 9.07 %
Average Artefacts = 11.11 %
Average Lumen = 11.36 %

Frames with Intestinal Mucosa under 15% = 175
Frames with Intestinal Mucosa between 15% and 50% = 757
Frames with Intestinal Mucosa between 50% and 75% = 771
Frames with Intestinal Mucosa over 75% = 1865

Frames with Residues under 5% = 2671
Frames with Residues between 5% and 25% = 381
Frames with Residues between 25% and 50% = 236
Frames with Residues over 50% = 280

Frames with Artefacts under 5% = 1068
Frames with Artefacts between 5% and 25% = 2227
Frames with Artefacts between 25% and 34% = 273
Frames with Artefacts over 34% = 0

Frames with Lumen under 5% = 1999
Frames with Lumen between 5% and 25% = 1025
Frames with Lumen between 25% and 50% = 382
Frames with Lumen over 50% = 162

Test Colonoscopy T7
9N 150 0.001 0.0007 0.00049
Artefacts under 34%

Total frames = 7664
Valid frames = 6231

Average Intestinal Mucosa = 63.72 %
Average Residues = 10.65 %
Average Artefacts = 16.86 %
Average Lumen = 8.76 %

Frames with Intestinal Mucosa under 15% = 79
Frames with Intestinal Mucosa between 15% and 50% = 1771
Frames with Intestinal Mucosa between 50% and 75% = 2166
Frames with Intestinal Mucosa over 75% = 2215

Frames with Residues under 5% = 2874
Frames with Residues between 5% and 25% = 2644
Frames with Residues between 25% and 50% = 655
Frames with Residues over 50% = 58

Frames with Artefacts under 5% = 517
Frames with Artefacts between 5% and 25% = 4422
Frames with Artefacts between 25% and 34% = 1292
Frames with Artefacts over 34% = 0

Frames with Lumen under 5% = 3389
Frames with Lumen between 5% and 25% = 2240
Frames with Lumen between 25% and 50% = 462
Frames with Lumen over 50% = 140

Segment #1
Total segment frames = 1500
Valid segment frames = 1145

Average Intestinal Mucosa = 40.64 %
Average Residues = 23.52 %
Average Artefacts = 22.04 %
Average Lumen = 13.80 %

Frames with Intestinal Mucosa under 15% = 49
Frames with Intestinal Mucosa between 15% and 50% = 930

Frames with Intestinal Mucosa between 50% and 75% = 148
Frames with Intestinal Mucosa over 75% = 18

Frames with Residues under 5% = 58
Frames with Residues between 5% and 25% = 697
Frames with Residues between 25% and 50% = 361
Frames with Residues over 50% = 29

Frames with Artefacts under 5% = 52
Frames with Artefacts between 5% and 25% = 666
Frames with Artefacts between 25% and 34% = 427
Frames with Artefacts over 34% = 0

Frames with Lumen under 5% = 132
Frames with Lumen between 5% and 25% = 891
Frames with Lumen between 25% and 50% = 64
Frames with Lumen over 50% = 58

Segment #2
Total segment frames = 2125
Valid segment frames = 1347

Average Intestinal Mucosa = 53.60 %
Average Residues = 11.54 %
Average Artefacts = 20.62 %
Average Lumen = 14.24 %

Frames with Intestinal Mucosa under 15% = 24
Frames with Intestinal Mucosa between 15% and 50% = 535
Frames with Intestinal Mucosa between 50% and 75% = 636
Frames with Intestinal Mucosa over 75% = 152

Frames with Residues under 5% = 464
Frames with Residues between 5% and 25% = 756
Frames with Residues between 25% and 50% = 112
Frames with Residues over 50% = 15

Frames with Artefacts under 5% = 108
Frames with Artefacts between 5% and 25% = 699
Frames with Artefacts between 25% and 34% = 540
Frames with Artefacts over 34% = 0

Frames with Lumen under 5% = 634
Frames with Lumen between 5% and 25% = 410
Frames with Lumen between 25% and 50% = 238

Frames with Lumen over 50% = 65

Segment #3
Total segment frames = 4039
Valid segment frames = 3739

Average Intestinal Mucosa = 74.44 %
Average Residues = 6.40 %
Average Artefacts = 13.92 %
Average Lumen = 5.24 %

Frames with Intestinal Mucosa under 15% = 6
Frames with Intestinal Mucosa between 15% and 50% = 306
Frames with Intestinal Mucosa between 50% and 75% = 1382
Frames with Intestinal Mucosa over 75% = 2045

Frames with Residues under 5% = 2352
Frames with Residues between 5% and 25% = 1191
Frames with Residues between 25% and 50% = 182
Frames with Residues over 50% = 14

Frames with Artefacts under 5% = 357
Frames with Artefacts between 5% and 25% = 3057
Frames with Artefacts between 25% and 34% = 325
Frames with Artefacts over 34% = 0

Frames with Lumen under 5% = 2623
Frames with Lumen between 5% and 25% = 939
Frames with Lumen between 25% and 50% = 160
Frames with Lumen over 50% = 17
